# (*Z*)-3-(4-Methoxy­anilino)-1-phenyl­but-2-en-1-one

**DOI:** 10.1107/S1600536809051186

**Published:** 2009-12-04

**Authors:** Li-Ping Zhang, Ming Yang, Yun Fang

**Affiliations:** a School of Chemical and Materials Engineering, Jiangnan University, 1800 Lihu Road, Wuxi 214122, Jiangsu, People’s Republic of China

## Abstract

In the title compound, C_17_H_17_NO_2_, the dihedral angle between the two benzene rings is 6.9 (1)°. The meth­oxy group is twisted slightly away from the aniline ring [C—O—C—C = 12.2 (3)°]. An intra­molecular N—H⋯O hydrogen bond generating an *S*(6) ring is observed. The crystal packing is stabilized by weak C—H⋯O and C—H⋯π inter­actions, forming a two-dimensional network.

## Related literature

For the biological activity of β-enamino ketones, see: Azzaro *et al.* (1981 ▶); Dannhardt *et al.* (1998 ▶); Boger *et al.* (1989 ▶); Wang *et al.* (1982 ▶). For the preparation of β-enamino ketones, see: Greenhill (1977 ▶); Elassar & El-Khair (2003 ▶); Zhang *et al.* (2006 ▶).
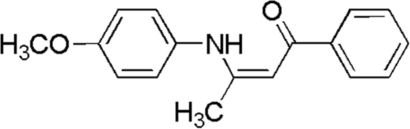


## Experimental

### 

#### Crystal data

C_17_H_17_NO_2_
*M* *_r_* = 267.32
Monoclinic, 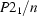

*a* = 6.435 (2) Å
*b* = 7.287 (3) Å
*c* = 30.919 (12) Å
β = 94.954 (6)°
*V* = 1444.5 (9) Å^3^
*Z* = 4
Mo *K*α radiation
μ = 0.08 mm^−1^
*T* = 294 K
0.24 × 0.20 × 0.16 mm

#### Data collection

Bruker SMART CCD area-detector diffractometer
Absorption correction: multi-scan (*SADABS*; Sheldrick, 1996 ▶) *T*_min_ = 0.739, *T*_max_ = 1.000
7729 measured reflections
2931 independent reflections
1900 reflections with *I* > 2σ(*I*)
*R*_int_ = 0.036

#### Refinement

*R*[*F* ^2^ > 2σ(*F* ^2^)] = 0.046
*wR*(*F* ^2^) = 0.135
*S* = 1.00
2931 reflections
184 parameters
H-atom parameters constrained
Δρ_max_ = 0.18 e Å^−3^
Δρ_min_ = −0.17 e Å^−3^

### 

Data collection: *SMART* (Bruker, 1998 ▶); cell refinement: *SAINT* (Bruker, 1999 ▶); data reduction: *SAINT*; program(s) used to solve structure: *SHELXS97* (Sheldrick, 2008 ▶); program(s) used to refine structure: *SHELXL97* (Sheldrick, 2008 ▶); molecular graphics: *SHELXTL* (Sheldrick, 2008 ▶); software used to prepare material for publication: *SHELXTL*.

## Supplementary Material

Crystal structure: contains datablocks global, I. DOI: 10.1107/S1600536809051186/ci2973sup1.cif

Structure factors: contains datablocks I. DOI: 10.1107/S1600536809051186/ci2973Isup2.hkl

Additional supplementary materials:  crystallographic information; 3D view; checkCIF report

## Figures and Tables

**Table 1:** Hydrogen-bond geometry (Å, °)

*D*—H⋯*A*	*D*—H	H⋯*A*	*D*⋯*A*	*D*—H⋯*A*
N1—H1⋯O2	0.86	1.91	2.629 (2)	139
C8—H8*B*⋯O2^i^	0.96	2.49	3.351 (3)	148
C3—H3⋯*Cg*2^ii^	0.93	2.84	3.712 (3)	156
C13—H13⋯*Cg*1^iii^	0.93	2.82	3.619 (3)	145

Symmetry codes: (i) 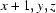; (ii) 
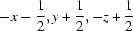
; (iii) 
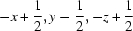
. *Cg*1 and *Cg*2 are the centroids of the C1–C6 and C12–C17 rings, respectively.

**Fig. 1.**
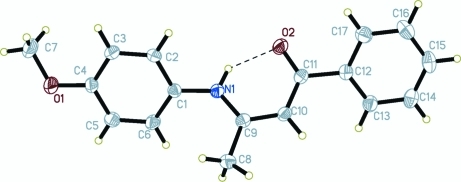
The molecular structure of the title compound, showing 30% probability displacement ellipsoids. The dashed line indicates a hydrogen bond.

**Fig. 2.**
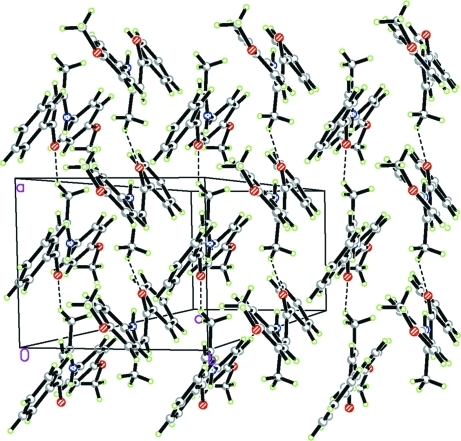
The crystal packing of the title compound, showing C—H···O hydrogen-bonded (dashed lines) chains along the a axis.

## supplementary crystallographic information

### Comment

β-enamino ketones have attracted considerable interest, because they are versatile intermediates for the synthesis of natural therapeutic and biologically active analogues including anticonvulsant (Azzaro *et al.*, 1981), anti-inflammatory (Dannhardt *et al.*, 1998) and antitumor agents (Boger *et al.*, 1989), as well as quinolone antibacterials (Wang *et al.*, 1982). It is therefore not surprising that many synthetic methods have been developed for the preparation of these compounds (Greenhill *et al.*, 1977; Elassar *et al.*, 2003). During the development of new environmental friendly methodologies (Zhang *et al.*, 2006) for the preparation of β-enamino ketones, we synthesized the title compound (Fig.1) and its crystal structure is reported here.

In the title compound, the dihedral angle between the two benzene rings is 6.9 (1)%. The methoxy group is slightly twisted away from the aniline ring, with a C7—O1—C4—C3 torsion angle of 12.2 (3)°. The C10—C11 bond length [1.415 (2) Å] is shorter than the C11—C12 bond length [1.500 (2) Å], and the N1—C9 bond length [1.333 (2) Å] is markedly shorter than the N1—C1 [1.419 (2) Å] bond length, indicating a strong electron delocalization. An intramolecular N1—H1···O2 hydrogen bond observed.

The crystal packing is stabilized by weak C—H···O and C—H···π interactions. Intermolecular C8—H8B···O2 hydrogen bonds link the molecules into a C(6) chain propagating along the *a* axis (Fig.2). In addition, the crystal packing is stabilized by C—H···π interactions; these interactions link the chains along the *b* axis, forming a two-dimensional network (Fig.2).

### Experimental

A mixture of 1-phenylbutane-1,3-dione (5 mmol), 4-methoxybenzenamine (5 mmol) and InBr_3_ (0.05 mmol) was stirred at room temperature for 1 h. After completion of the reaction, the reaction mixture was diluted with H_2_O (10 ml) and extracted with EtOAc (210 ml). The combined organic layers were dried, concentrated, purified by column chromatography on SiO_2_ with ethyl acetate-cyclohexane (2:8), to obatin a pale yellow solid, with a yield of 78% (m. p. 84–85° C); IR (neat):ν 2979, 2870,1608, 1576, 1504, 1473, 1432,1372, 820, 744 cm^-1^; ^1^H NMR(CDCl_3_, 300 MHz): δ 2.06(s, 3H), 3.80(s, 3H), 5.86(s, 1H), 6.88(d, 2H,Ar—H), 7.09(d, 2H,Ar—H), 7.42–7.45(m, 3H,Ph), 7.89–7.92 (m, 2H, Ph), 12.92 (br s, 1H, NH). ^13^C NMR(CDCl_3_, 75 MHz): δ 20.2, 63.7, 93.5, 114.8, 126.5, 127.0, 128.2, 130.7, 131.3, 140.1, 157.2, 163.1, 188.3. ESI-MS: 268(*M*+1)^+^. Analysis calculated for C_17_H_17_NO_2_: C 76.38, H 6.41, N 5.24; found: C 76.53, H 6.52, N 5.32. Single crystals suitable for X-ray diffraction study were obtained from ethyl acetate-cyclohexane by slow evaporation at room temperature.

### Refinement

H atoms were placed in geometrically idealized positions and constrained to ride on their parent atoms, with N-H = 0.86 Å, C-H = 0.93–0.96 Å, and *U*_iso_(H) = 1.5*U*_eq_(CH_3_) or 1.2*U*_eq_(C,N). Each methyl group was allowed to rotate freely about its C—C bond.

### Crystal data

**Table:**

C_17_H_17_NO_2_	*F*(000) = 568
*M**_r_* = 267.32	*D*_x_ = 1.229 Mg m^−^^3^
Monoclinic, *P*2_1_/*n*	Mo *K*α radiation, λ = 0.71073 Å
Hall symbol: -P 2yn	Cell parameters from 2333 reflections
*a* = 6.435 (2) Å	θ = 2.6–26.3°
*b* = 7.287 (3) Å	µ = 0.08 mm^−^^1^
*c* = 30.919 (12) Å	*T* = 294 K
β = 94.954 (6)°	Block, yellow
*V* = 1444.5 (9) Å^3^	0.24 × 0.20 × 0.16 mm
*Z* = 4	

### Data collection

**Table:**

Bruker SMART CCD area-detector diffractometer	2931 independent reflections
Radiation source: fine-focus sealed tube	1900 reflections with *I* > 2σ(*I*)
graphite	*R*_int_ = 0.036
φ and ω scans	θ_max_ = 26.4°, θ_min_ = 1.3°
Absorption correction: multi-scan (*SADABS*; Sheldrick, 1996)	*h* = −4→8
*T*_min_ = 0.739, *T*_max_ = 1.000	*k* = −9→7
7729 measured reflections	*l* = −34→38

### Refinement

**Table:**

Refinement on *F*^2^	Secondary atom site location: difference Fourier map
Least-squares matrix: full	Hydrogen site location: inferred from neighbouring sites
*R*[*F*^2^ > 2σ(*F*^2^)] = 0.046	H-atom parameters constrained
*wR*(*F*^2^) = 0.135	*w* = 1/[σ^2^(*F*_o_^2^) + (0.0645*P*)^2^ + 0.2627*P*] where *P* = (*F*_o_^2^ + 2*F*_c_^2^)/3
*S* = 1.00	(Δ/σ)_max_ = 0.001
2931 reflections	Δρ_max_ = 0.18 e Å^−^^3^
184 parameters	Δρ_min_ = −0.17 e Å^−^^3^
0 restraints	Extinction correction: *SHELXL97* (Sheldrick, 2008), Fc^*^=kFc[1+0.001xFc^2^λ^3^/sin(2θ)]^-1/4^
Primary atom site location: structure-invariant direct methods	Extinction coefficient: 0.035 (3)

### Special details

**Table:**

Geometry. All e.s.d.'s (except the e.s.d. in the dihedral angle between two l.s. planes) are estimated using the full covariance matrix. The cell e.s.d.'s are taken into account individually in the estimation of e.s.d.'s in distances, angles and torsion angles; correlations between e.s.d.'s in cell parameters are only used when they are defined by crystal symmetry. An approximate (isotropic) treatment of cell e.s.d.'s is used for estimating e.s.d.'s involving l.s. planes.
Refinement. Refinement of *F*^2^ against ALL reflections. The weighted *R*-factor *wR* and goodness of fit *S* are based on *F*^2^, conventional *R*-factors *R* are based on *F*, with *F* set to zero for negative *F*^2^. The threshold expression of *F*^2^ > σ(*F*^2^) is used only for calculating *R*-factors(gt) *etc*. and is not relevant to the choice of reflections for refinement. *R*-factors based on *F*^2^ are statistically about twice as large as those based on *F*, and *R*- factors based on ALL data will be even larger.

### Fractional atomic coordinates and isotropic or equivalent isotropic displacement parameters (Å^2^)

**Table:**

	*x*	*y*	*z*	*U*_iso_*/*U*_eq_	
O1	0.0332 (2)	0.1669 (2)	0.45901 (4)	0.0621 (4)	
O2	−0.17087 (19)	0.1729 (2)	0.20705 (4)	0.0552 (4)	
N1	0.0723 (2)	0.1693 (2)	0.27987 (4)	0.0417 (4)	
H1	−0.0489	0.1722	0.2658	0.050*	
C1	0.0705 (2)	0.1739 (2)	0.32573 (5)	0.0364 (4)	
C2	−0.0759 (3)	0.2824 (3)	0.34338 (5)	0.0412 (4)	
H2	−0.1639	0.3558	0.3253	0.049*	
C3	−0.0940 (3)	0.2835 (3)	0.38775 (5)	0.0445 (5)	
H3	−0.1947	0.3560	0.3992	0.053*	
C4	0.0378 (3)	0.1768 (2)	0.41473 (5)	0.0423 (4)	
C5	0.1869 (3)	0.0706 (3)	0.39734 (6)	0.0515 (5)	
H5	0.2780	0.0005	0.4156	0.062*	
C6	0.2022 (3)	0.0673 (3)	0.35319 (6)	0.0480 (5)	
H6	0.3015	−0.0069	0.3418	0.058*	
C7	−0.1412 (4)	0.2438 (4)	0.47712 (6)	0.0798 (8)	
H7A	−0.1385	0.3749	0.4740	0.120*	
H7B	−0.1366	0.2126	0.5074	0.120*	
H7C	−0.2670	0.1963	0.4623	0.120*	
C8	0.4516 (3)	0.1682 (3)	0.27522 (6)	0.0504 (5)	
H8A	0.4579	0.2429	0.3009	0.076*	
H8B	0.5399	0.2199	0.2549	0.076*	
H8C	0.4981	0.0463	0.2828	0.076*	
C9	0.2316 (3)	0.1612 (2)	0.25509 (5)	0.0384 (4)	
C10	0.1947 (3)	0.1546 (2)	0.21040 (5)	0.0397 (4)	
H10	0.3087	0.1449	0.1940	0.048*	
C11	−0.0069 (3)	0.1616 (2)	0.18819 (5)	0.0386 (4)	
C12	−0.0272 (3)	0.1643 (2)	0.13949 (5)	0.0390 (4)	
C13	0.1114 (3)	0.0745 (3)	0.11502 (5)	0.0485 (5)	
H13	0.2218	0.0086	0.1288	0.058*	
C14	0.0869 (3)	0.0820 (3)	0.07020 (6)	0.0592 (6)	
H14	0.1797	0.0195	0.0540	0.071*	
C15	−0.0728 (4)	0.1807 (3)	0.04946 (6)	0.0612 (6)	
H15	−0.0872	0.1869	0.0193	0.073*	
C16	−0.2117 (4)	0.2703 (3)	0.07320 (6)	0.0621 (6)	
H16	−0.3203	0.3375	0.0591	0.075*	
C17	−0.1908 (3)	0.2612 (3)	0.11809 (6)	0.0525 (5)	
H17	−0.2871	0.3205	0.1340	0.063*	

### Atomic displacement parameters (Å^2^)

**Table:**

	*U*^11^	*U*^22^	*U*^33^	*U*^12^	*U*^13^	*U*^23^
O1	0.0685 (10)	0.0814 (11)	0.0370 (7)	0.0231 (8)	0.0080 (6)	0.0056 (7)
O2	0.0377 (7)	0.0836 (11)	0.0449 (7)	−0.0037 (7)	0.0074 (6)	−0.0087 (7)
N1	0.0333 (8)	0.0548 (10)	0.0370 (8)	−0.0018 (7)	0.0037 (6)	−0.0034 (7)
C1	0.0366 (9)	0.0376 (10)	0.0348 (9)	−0.0016 (7)	0.0027 (7)	−0.0008 (7)
C2	0.0355 (9)	0.0476 (11)	0.0399 (10)	0.0068 (8)	−0.0001 (7)	0.0040 (8)
C3	0.0426 (10)	0.0488 (12)	0.0429 (10)	0.0107 (8)	0.0082 (8)	−0.0013 (8)
C4	0.0476 (10)	0.0462 (11)	0.0329 (9)	0.0030 (8)	0.0031 (8)	0.0010 (8)
C5	0.0565 (12)	0.0516 (12)	0.0463 (11)	0.0195 (10)	0.0038 (9)	0.0093 (9)
C6	0.0557 (11)	0.0438 (11)	0.0456 (11)	0.0178 (9)	0.0100 (8)	0.0023 (9)
C7	0.0960 (18)	0.102 (2)	0.0442 (12)	0.0378 (15)	0.0206 (12)	0.0024 (12)
C8	0.0375 (10)	0.0637 (13)	0.0498 (11)	0.0002 (9)	0.0039 (8)	0.0037 (9)
C9	0.0368 (9)	0.0355 (10)	0.0434 (10)	−0.0023 (7)	0.0066 (7)	0.0012 (8)
C10	0.0372 (9)	0.0442 (11)	0.0388 (9)	−0.0009 (8)	0.0098 (7)	0.0003 (8)
C11	0.0404 (10)	0.0363 (10)	0.0398 (9)	−0.0036 (8)	0.0082 (8)	−0.0024 (8)
C12	0.0424 (10)	0.0361 (10)	0.0384 (9)	−0.0062 (8)	0.0023 (7)	−0.0024 (8)
C13	0.0552 (12)	0.0484 (12)	0.0425 (11)	0.0028 (9)	0.0071 (8)	−0.0012 (9)
C14	0.0729 (14)	0.0622 (14)	0.0439 (11)	−0.0019 (11)	0.0137 (10)	−0.0078 (10)
C15	0.0862 (16)	0.0605 (14)	0.0358 (10)	−0.0136 (12)	−0.0008 (10)	−0.0006 (10)
C16	0.0730 (14)	0.0571 (14)	0.0526 (12)	0.0022 (11)	−0.0148 (10)	0.0031 (10)
C17	0.0555 (12)	0.0505 (12)	0.0505 (12)	0.0040 (10)	−0.0016 (9)	−0.0073 (10)

### Geometric parameters (Å, °)

**Table:**

O1—C4	1.374 (2)	C8—C9	1.497 (2)
O1—C7	1.413 (2)	C8—H8A	0.96
O2—C11	1.251 (2)	C8—H8B	0.96
N1—C9	1.333 (2)	C8—H8C	0.96
N1—C1	1.419 (2)	C9—C10	1.383 (2)
N1—H1	0.86	C10—C11	1.415 (2)
C1—C2	1.378 (2)	C10—H10	0.93
C1—C6	1.385 (2)	C11—C12	1.500 (2)
C2—C3	1.387 (2)	C12—C13	1.383 (2)
C2—H2	0.93	C12—C17	1.387 (3)
C3—C4	1.378 (2)	C13—C14	1.382 (2)
C3—H3	0.93	C13—H13	0.93
C4—C5	1.378 (2)	C14—C15	1.368 (3)
C5—C6	1.377 (2)	C14—H14	0.93
C5—H5	0.93	C15—C16	1.370 (3)
C6—H6	0.93	C15—H15	0.93
C7—H7A	0.96	C16—C17	1.385 (3)
C7—H7B	0.96	C16—H16	0.93
C7—H7C	0.96	C17—H17	0.93
			
C4—O1—C7	117.43 (15)	C9—C8—H8C	109.5
C9—N1—C1	130.43 (15)	H8A—C8—H8C	109.5
C9—N1—H1	114.8	H8B—C8—H8C	109.5
C1—N1—H1	114.8	N1—C9—C10	120.13 (16)
C2—C1—C6	118.82 (15)	N1—C9—C8	120.40 (15)
C2—C1—N1	118.30 (15)	C10—C9—C8	119.40 (15)
C6—C1—N1	122.79 (15)	C9—C10—C11	123.68 (15)
C1—C2—C3	120.92 (16)	C9—C10—H10	118.2
C1—C2—H2	119.5	C11—C10—H10	118.2
C3—C2—H2	119.5	O2—C11—C10	123.41 (16)
C4—C3—C2	119.71 (16)	O2—C11—C12	117.59 (15)
C4—C3—H3	120.1	C10—C11—C12	118.94 (14)
C2—C3—H3	120.1	C13—C12—C17	118.60 (16)
O1—C4—C5	115.81 (15)	C13—C12—C11	122.58 (16)
O1—C4—C3	124.58 (16)	C17—C12—C11	118.82 (16)
C5—C4—C3	119.61 (16)	C14—C13—C12	120.45 (18)
C6—C5—C4	120.56 (17)	C14—C13—H13	119.8
C6—C5—H5	119.7	C12—C13—H13	119.8
C4—C5—H5	119.7	C15—C14—C13	120.44 (19)
C5—C6—C1	120.36 (17)	C15—C14—H14	119.8
C5—C6—H6	119.8	C13—C14—H14	119.8
C1—C6—H6	119.8	C14—C15—C16	119.87 (18)
O1—C7—H7A	109.5	C14—C15—H15	120.1
O1—C7—H7B	109.5	C16—C15—H15	120.1
H7A—C7—H7B	109.5	C15—C16—C17	120.2 (2)
O1—C7—H7C	109.5	C15—C16—H16	119.9
H7A—C7—H7C	109.5	C17—C16—H16	119.9
H7B—C7—H7C	109.5	C16—C17—C12	120.42 (18)
C9—C8—H8A	109.5	C16—C17—H17	119.8
C9—C8—H8B	109.5	C12—C17—H17	119.8
H8A—C8—H8B	109.5		
			
C9—N1—C1—C2	142.48 (19)	N1—C9—C10—C11	2.1 (3)
C9—N1—C1—C6	−40.9 (3)	C8—C9—C10—C11	−175.06 (16)
C6—C1—C2—C3	−0.9 (3)	C9—C10—C11—O2	−0.6 (3)
N1—C1—C2—C3	175.86 (16)	C9—C10—C11—C12	176.46 (16)
C1—C2—C3—C4	0.9 (3)	O2—C11—C12—C13	−149.89 (18)
C7—O1—C4—C5	−167.8 (2)	C10—C11—C12—C13	32.8 (2)
C7—O1—C4—C3	12.2 (3)	O2—C11—C12—C17	30.3 (2)
C2—C3—C4—O1	−179.76 (17)	C10—C11—C12—C17	−146.95 (18)
C2—C3—C4—C5	0.3 (3)	C17—C12—C13—C14	0.2 (3)
O1—C4—C5—C6	178.64 (18)	C11—C12—C13—C14	−179.60 (17)
C3—C4—C5—C6	−1.4 (3)	C12—C13—C14—C15	1.0 (3)
C4—C5—C6—C1	1.4 (3)	C13—C14—C15—C16	−1.1 (3)
C2—C1—C6—C5	−0.2 (3)	C14—C15—C16—C17	0.0 (3)
N1—C1—C6—C5	−176.83 (17)	C15—C16—C17—C12	1.2 (3)
C1—N1—C9—C10	178.77 (16)	C13—C12—C17—C16	−1.3 (3)
C1—N1—C9—C8	−4.1 (3)	C11—C12—C17—C16	178.51 (17)

### Hydrogen-bond geometry (Å, °)

**Table:**

*D*—H···*A*	*D*—H	H···*A*	*D*···*A*	*D*—H···*A*
N1—H1···O2	0.86	1.91	2.629 (2)	139
C8—H8B···O2^i^	0.96	2.49	3.351 (3)	148
C3—H3···Cg2^ii^	0.93	2.84	3.712 (3)	156
C13—H13···Cg1^iii^	0.93	2.82	3.619 (3)	145

Symmetry codes: (i) *x*+1, *y*, *z*; (ii) −*x*−1/2, *y*+1/2, −*z*+1/2; (iii) −*x*+1/2, *y*−1/2, −*z*+1/2.
